# WHAT DO DIRECT CARE WORKERS NEED TRAINING IN TO HELP OLDER ADULTS WITH DEMENTIA DURING MEALTIMES?

**DOI:** 10.1093/geroni/igae098.2282

**Published:** 2024-12-31

**Authors:** Dukyoo Jung, Jisung Park, Eunju Choi, Leeho Yoo, Kahyun Kim, Seyoung Cho, Soogyung Shin

**Affiliations:** Ewha Womans University, Seoul, Republic of Korea; Ewha Womans University, Seoul, Republic of Korea; Ewha Womans University, Seoul, Republic of Korea; Ewha Womans University, Seoul, Republic of Korea; Ewha Womans University, Seoul, Republic of Korea; Ewha Womans University, Seoul, Republic of Korea; Ewha Womans University, Seoul, Republic of Korea

## Abstract

Older adults with dementia in long-term care facilities often encounter challenges in self-feeding owing to cognitive and physical impairments. Although direct care workers play a crucial role in facilitating meal-time activities, they lack adequate training and specific educational programs. To develop an effective program, it is essential to understand the educational needs of direct care workers providing meal assistance. This study aimed to identify the educational needs of direct care workers in long-term care facilities regarding providing meal assistance for older adults with dementia. Adopting a mixed-methods approach, this study combined quantitative analysis, using Borich’s needs assessment, and the locus for focus models, with qualitative insights from focus group interviews. Participants comprised 175 direct care workers and 5 nursing managers from various long-term care facilities in South Korea. This study identified four main educational priorities: enhancing knowledge of swallowing functions, understanding institutional support mechanisms, applying multisensory stimulation techniques, and addressing food forgetfulness in older adults with dementia. These findings were supported by qualitative data that emphasized the necessity of training to improve the quality of meal assistance provided to this vulnerable population. The findings underscore the critical need for focused educational programs that equip direct care workers with the skills and knowledge necessary to effectively assist older adults with dementia during mealtime. This study advocates the implementation of continuous education and training initiatives led by nursing management to foster an environment conducive to autonomous and independent eating among patients with dementia, thereby enhancing their overall care and quality of life.

